# One Size Does Not Fit All: Achieving Trachoma Control by 2030

**DOI:** 10.4269/ajtmh.19-0684

**Published:** 2019-10-07

**Authors:** Catherine E. Oldenburg

**Affiliations:** Francis I Proctor Foundation Department of Ophthalmology and Epidemiology and Biostatistics, University of California, San Francisco, California

The WHO has the ambitious goal of controlling trachoma as a public health problem by the year 2020, a target which will not be met.^1,2^ Trachoma control is defined by the prevalence of trachomatous inflammation—follicular (TF) and trachomatous trichiasis (TT) at the health district level estimated from population-based surveys. The current elimination thresholds are TF prevalence of < 5% in children aged 1–9 years and TT prevalence of < 1 case unknown to the health system (as of yet without consideration for surgery) per 1,000 persons. The WHO strategy for achieving trachoma control, referred to as the “SAFE strategy,” is a multifaceted approach aimed to address multiple transmission routes and stages of the epidemic. “S” refers to surgery for trichiasis, to reduce the risk of developing corneal opacity in individuals who had trachoma during childhood. “A” refers to annual mass distribution of azithromycin to entire communities where trachoma is endemic to control transmission of the ocular strains of *Chlamydia trachomatis* that cause trachoma. “F” and “E” refer to facial cleanliness and environmental improvements, such as increasing access to safe water and latrinization, meant to aid with control of the fly vector *Musca sorbens* and reduce ocular chlamydia transmission. Implementation of the SAFE strategy is recommended in districts with TF prevalence greater than 10%, with the duration of implementation varying based on TF prevalence from trachoma impact surveys. Although recommendations for the number of years of intervention vary by TF prevalence, the content of the A, F, and E components do not vary in a given year in districts with TF prevalence above the threshold.

Whereas the trachoma control program has had remarkable success in many formerly endemic regions, two reports in this issue of the *American Journal of Tropical Medicine and Hygiene* describe the heterogeneous current epidemiology of trachoma. Sanders et al.^3^ present compelling data suggesting that Sudan is close to trachoma elimination. Stewart et al.^4^ present data from Amhara, Ethiopia, demonstrating persistently high levels of TF in some districts, despite many years of implementation of the SAFE strategy. These data describe two very different trachoma epidemics. In Sudan, low TF prevalence indicates that the epidemic will disappear, perhaps even without additional intervention. Conversely, in Amhara, elimination of trachoma in the next decade may be an unrealistic goal without a change in strategy. These data suggest that TF-based strategies for trachoma control will need to be tailored to local contexts.

Although the WHO 2020 target will not be met, incredible progress has been made. The number of people living in areas with endemic trachoma has declined by 91% since 2002.^2^ Many of the remaining districts where TF prevalence remains greater than 5% are similar to those described in Sudan.^3^ In districts with low prevalence of TF that is not yet less than 5%, continued implementation of SAFE is indicated as per the present guidelines, but it may not be necessary to eliminate infection. TF is a lagging indicator for ocular chlamydia infection, which may persist in communities after chlamydia transmission has ceased.^5^ Based on experience in many areas, in communities with TF prevalence close to the control threshold, any remaining TF will likely disappear. Furthermore, if there is no true chlamydia transmission ongoing in low TF-prevalent communities, additional rounds of azithromycin distribution will not help reduce the prevalence of trachoma. Even without additional intervention, these districts will likely achieve control well within the next decade.

The challenge for trachoma control now rests with persistent infection in a few districts, most of which are in Amhara, Ethiopia.^4,5^ Annual mass drug administration with azithromycin, alongside scale-up of water, sanitation, and hygiene (WASH) interventions, has been ongoing in many of these districts for over 10 years. However, many districts still remain far from achieving control, and, following the present guidelines, achieving control will likely take many additional years. Achieving control more quickly will require a change in strategy. Current options include further scale-up of WASH or more intensive antibiotics. Although facial cleanliness and environmental improvements are core components of the trachoma control strategy, their efficacy has yet to be established in a randomized controlled trial^6,7^ and it remains unclear what impact, if any, intensified WASH will have for trachoma control efforts. Intensified antibiotic treatment, such as treating all children quarterly, is the only strategy to date that has been shown to perform significantly better than annual mass azithromycin distribution.^8^ Although such a strategy would not have been logistically feasible 10 years ago, when proof of concept studies were completed, today there are a much more limited number of districts in which programs will need to focus their efforts. A shift in focus to more intensified antibiotic treatment and away from strategies lacking a rigorous evidence base could save program resources in the long run, by reducing longer term antibiotic distribution and decreasing time until control. Such an achievement would both reduce the risk of avoidable blindness due to trachoma and reduce unnecessary risk of selection for antimicrobial resistance from mass distribution of antibiotics.^9^

How can we maximize the probability that trachoma is eliminated in the next decade? As trachoma approaches control targets, two distinct epidemiologic situations are emerging. In the vast majority of affected districts, trachoma is disappearing and may continue to do so without further intervention. In a limited number of remaining hot spots, more intensified intervention will likely be required to eliminate infection. Presently, our best option may be to scale-up intensified azithromycin distribution in districts with persistent infection while scaling down activities in districts that are approaching control. The epidemic has reached the stage where tailoring interventions to suit the local epidemiology is required, and a one-size-fits-all approach will no longer suffice.
